# Pachydermodactyly: An Underdiagnosed Condition in Adolescence—A Case Report and Literature Review

**DOI:** 10.1155/crdm/5560071

**Published:** 2025-05-08

**Authors:** Mishari T. Alrubaiaan, Yousef H. Alharthi, Suliman Alfaraj

**Affiliations:** ^1^College of Medicine, King Saud Bin Abdulaziz University for Health Sciences, Riyadh, Saudi Arabia; ^2^Department of Dermatology and Dermatologic Surgery, Prince Sultan Military Medical City, Riyadh, Saudi Arabia

## Abstract

Pachydermodactyly (PDD) is a rare, underdiagnosed benign condition characterized by asymmetrical, bilateral fusiform swellings of the hands' proximal interphalangeal (PIP) joints. In this type of digital fibromatosis, cutaneous thickening is thought to occur due to repetitive mechanical irritation. Furthermore, due to its striking clinical appearance, PDD is commonly overlooked or misdiagnosed as other inflammatory arthropathies or pachydermoperiostosis. In addition, because of its elusive nature and resemblance to more serious conditions, clinicians should be aware of this condition. Herein, we present a case of PDD and discuss the differential diagnoses to improve recognition and prevent misdiagnosis.

## 1. Introduction

Pachydermodactyly (PDD), derived from the Greek words “pachy” (thick), “dermis” (skin), and “dactylos” (finger), is a rare, benign, and asymptomatic form of cutaneous fibromatosis [1]. It was first classified as knuckle pads by Verbov in 1975 [1]. As a distinct clinical entity, it is characterized by painless, nonpruritic soft tissue swelling of the lateral aspect of the proximal interphalangeal (PIP) joint, resulting in fusiform or saccular swellings on both the radial and ulnar aspects [2]. Despite its benign nature, a lack of awareness can lead to unnecessary investigations and treatments [3]. This report presents a case of PDD that was promptly recognized and appropriately managed.

## 2. Case Presentation

**Figure 1:**
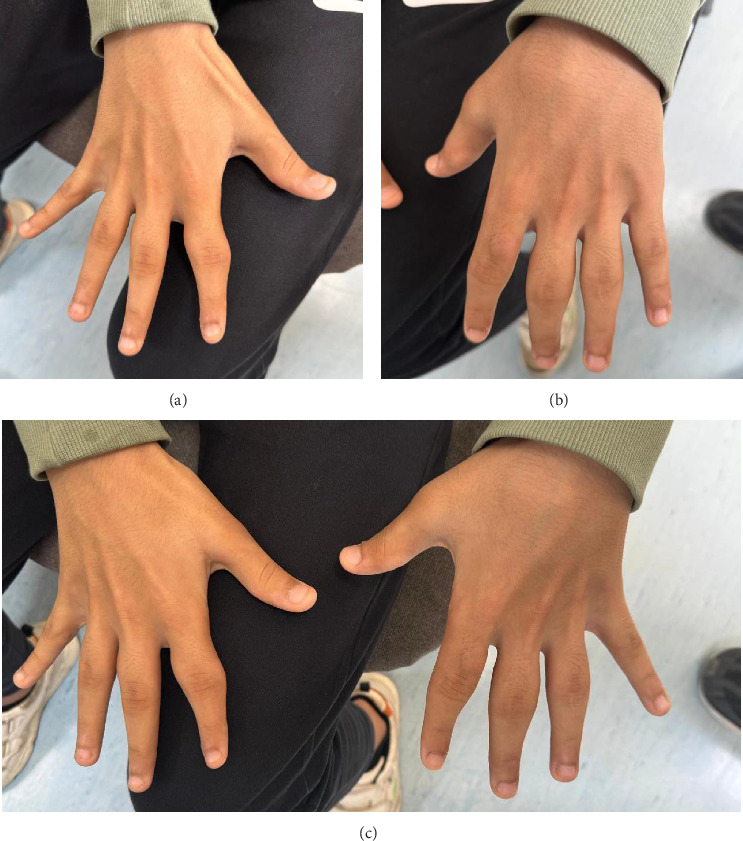
Clinical photographs of the right (a), left (b), and both hands (c) demonstrate symmetrical fusiform swelling involving the proximal interphalangeal joints of the second to fourth digits on both hands.

A 14-year-old male with a known case of hyperopic astigmatism and autoimmune pancreatitis maintained on *azathioprine* 50 mg PO for 8 years. The patient presented to our dermatology clinic complaining of a 2-year history of progressive, symmetrical, painless, nonpruritic swelling of the soft tissue around the PIP joints of the 2^nd^, 3^rd^, and 4^th^ fingers bilaterally (Figure 1). He reported using PlayStation controller and mobile games repetitively, on average 5 h per day. He denied having morning stiffness, fever, rash, mouth ulcers, uveitis, or gastrointestinal symptoms. He remained active in his activities and without any loss of function reported. In addition, he denied having any personal history of a preceding traumatic event or psychiatric disorders. Furthermore, he denied having a family history of rheumatologic conditions. However, his mother and brother are carriers of an ADAMTS-13 mutation while genetic testing confirmed that our patient does not carry this mutation.

Physical examination revealed symmetrical soft tissue swelling on the PIP joints of the 2^nd^, 3^rd^, and 4^th^ fingers bilaterally (Figure 1), with the left hand being affected more than the right hand (Figure 1). The patient was, otherwise, asymptomatic, and specifically denied having any pruritus, tenderness, pain, and warmth or burning sensation in his fingers. Moreover, the range of motion was within normal range and no other joints were affected, and no skin or nail changes were observed.

**Figure 2:**
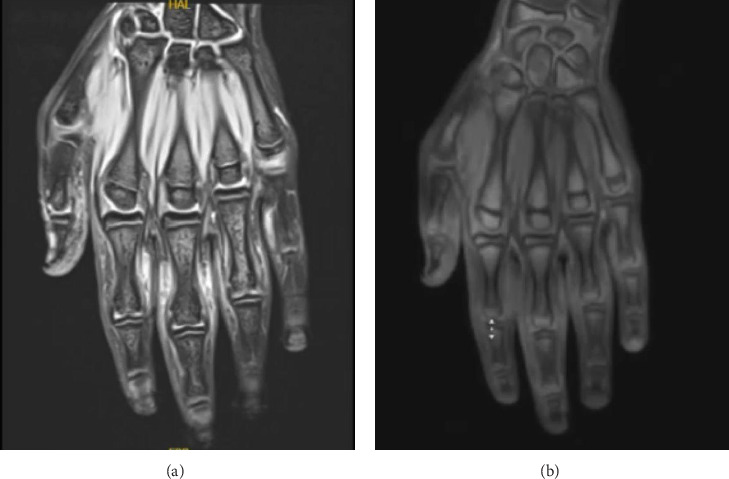
Coronal views (a-b) of magnetic resonance imaging of the left hand reveal an oval-shaped subcutaneous soft tissue mass, characterized by T1 hypointensity and minimally enhancing T2 hyperintensity, with no evidence of joint effusion or synovitis.

The results of the laboratory investigations, including a complete blood count, liver and renal function tests, erythrocyte sedimentation rate, C-reactive protein, rheumatoid factor, and antinuclear antibody were within normal limits or negative. Furthermore, magnetic resonance imaging of the left hand only noted a subcutaneous oval-shaped soft tissue mass, which showed a T1 hypointensity and a minimally enhancing T2 hyperintensity with no joint effusion or synovitis (Figure 2). Histopathologic analysis was not performed, and the patient was diagnosed with PDD. The patient swelling was mainly attributed to repetitive mechanical trauma from extensive use of gaming controllers, so by recognizing the potential impact of such habitual behaviors we initially recommended behavioral modifications aiming at reducing mechanical stress on affected digits. Moreover, he was offered an intralesional steroid injection; however, the patient preferred to be referred to plastic surgery for possible surgical excision.

## 3. Discussion

Since Verbov's initial report in 1975 [1], cases of PDD have been documented worldwide. However, despite increased reporting, the condition remains rare and likely underdiagnosed, as the low number of reported cases may not reflect its true prevalence. In addition, PDD is often misdiagnosed as an inflammatory arthropathy due to its overlapping clinical features [1].

PDD has been recognized as a distinct clinical entity due to its typical presentation as painless, nonpruritic thickening of the lateral side of the PIP joint, resulting in fusiform or saccular swellings on the radial and ulnar sides [2]. The second through fourth digits are usually affected bilaterally, with occasional involvement of the fifth digit [2]. The soft tissue swelling tends to evolve insidiously and symmetrically, as observed in our patient who presented with a slowly progressive symmetrical swelling of the PIP joints [2–4]. Furthermore, although PDD is characterized by absence of joint involvement, pain, or functional loss, there was one reported case of deformable PDD with nonerosive subluxation of the interphalangeal joint, constituting an atypical presentation [5]. However, our patient denied having pain or loss of function. In addition, our patient, a 14-year-old male in puberty, aligns with Dallos et al.'s observation of a median age of 14 for the onset of PDD [2], highlighting the typical age-related onset of this condition.

Even though the underlying etiology of PDD is unknown, there is evidence that mechanical irritation of the periarticular skin is a major contributor to the development of the condition [2]. Moreover, although PDD is a benign condition with minimal systemic implications, rare associations that are related to genetic predispositions and psychiatric conditions have been reported (Table 1). For instance, Ulusoy et al. reported a familial case of unilateral PDD, highlighting the potential role of hereditary factors in its development [21]. Similarly, Cabanillas et al. and Woodrow et al. linked PDD with psychiatric conditions such as compulsive behavioral habits, Asperger syndrome, and other neuropsychiatric disorders [23, 25]. Hence, this indicates the need for clinicians to assess and address potential underlying psychological conditions when diagnosing PDD and possibly integrating habit reversal training when managing PDD. In addition to behavioral and genetic associations, occupational and environmental factors have also been implicated in PDD. Sagransky et al. reported cases of PDD in poultry processing workers due to repetitive occupational motions, reinforcing the role of mechanical irritation in its pathogenesis [19]. Similarly, Chen et al. identified links between PDD and local trauma, such as knuckle cracking [20]. These findings align with our case, where the patient's extensive gaming habits likely contributed to the condition by repetitive mechanical irritation. Recognizing these environmental triggers is essential in managing PDD (Table 1).

The diagnosis of PDD is primarily clinical [1]. Laboratory markers are typically not elevated in blood tests [1]. Histopathologic examination can be valuable in differentiating PDD from other clinical entities; however, in our case, the clinical manifestation was characteristic of PDD. Hence, we relied on clinical and radiological findings. In addition, the literature shows that histopathologic examination is not often required in order to diagnose PDD, and the histopathological findings are often nonspecific [6, 10]. Moreover, X-rays may reveal soft tissue swelling and MRI can reveal swelling of soft tissues without synovitis, tendonitis, or joint effusion.

When it comes to the management of PDD, discontinuing mechanical stimulation in order to prevent progression and promote regression, or addressing underlying mental disorders, is the first option to consider [13]. Surgical excision or intralesional steroid injections can be effective in some cases [7, 9, 24].

## 4. Conclusion

Progressive symmetrical bilateral swelling of the PIP, in an otherwise healthy young male, should prompt the consideration of PDD in the differential diagnosis as it can be mistaken with other rheumatic conditions that present with digital bulbous swelling. A prompt diagnosis would prevent unnecessary investigations, reassure the patient, and avoid inappropriate treatment.

## Figures and Tables

**Table 1:** Literature review of included studies and sample characteristics.

Sr. no.	Author(s) and year	Study title	Case description	Age/gender	Sample size	Diagnostic findings	Differential diagnosis	Management	Outcomes	Reference
1	Ito et al. (2024)	“Symptomatic pachydermodactyly: a case report”	Symptomatic PDD in a young male requiring differentiation from JIA	14/male	1	Normal inflammatory markers, absence of uveitis, no bone destruction	Rheumatoid factor-negative polyarticular JIA	Observation; surgery for esthetic reasons	Morning stiffness resolved; swelling reduced	[6]
2	Gallardo-Villamil et al. (2023)	“Pachydermodactyly: Soft tissue enlargement of the fingers in a teenager”	Progressive asymptomatic soft tissue enlargement in a teenager with histological confirmation of PDD	16/male	1	Normal serological tests, soft tissue swelling on X-ray, histopathology showing collagen, and mucin deposition	Juvenile idiopathic arthritis, rheumatoid arthritis	Intralesional corticosteroids	Mild improvement in swelling and functionality	[7]
3	Tariq et al. (2023)	“Pachydermodactyly, mimicker of rheumatoid hands, presents in a patient with tuberous sclerosis”	Case of PDD coexisting with tuberous sclerosis presenting as symmetrical joint swellings	21/male	1	Symmetrical MCP and PIP joint swelling; negative serology; X-ray showing soft tissue swelling without erosion	Rheumatoid arthritis, psoriatic arthritis, multicentric reticulohistiocytosis	Intralesional steroids, surgical excision	Swelling reduced; improved hand grip and joint flexion	[4]
4	Aljohani (2022)	“Unilateral pachydermodactyly misdiagnosed as juvenile idiopathic arthritis: A case report”	Unilateral PDD in a young male misdiagnosed with JIA and treated with methotrexate for a year	16/male	1	Normal serological tests, X-ray showing soft tissue swelling, skin biopsy confirming collagen thickening	Juvenile idiopathic arthritis, Thiemann disease	Stopped methotrexate; observation advised	Symptoms stable; no progression after cessation of treatment	[8]
5	Sakai et al. (2022)	“Surgical management of pachydermodactyly (PDD) via midaxial incision: A case report”	Asymptomatic PIP joint swelling on both hands; confirmed diagnosis via surgical excision	15/male	1	Soft tissue swelling on MRI; no bone or joint involvement; histopathology confirmed PDD	Rheumatoid arthritis, connective tissue diseases	Surgical resection via midaxial incision with Z-plasty	Improved appearance; no recurrence or complications	[9]
6	Hussain et al. (2021)	“Painful pachydermodactyly in a 39-year-old woman: A case report and review of the literature”	Chronic symmetrical polyarticular pain with progressive swelling of PIP and DIP joints	39/female	1	Soft tissue swelling on X-ray; negative serology including RF, anti-CCP, and ANA	Rheumatoid arthritis, psoriatic arthritis, osteoarthritis	Vitamin D supplementation, etoricoxib, behavioral modification	Pain and swelling alleviated; stable condition	[10]
7	Novais et al. (2021)	“Pachydermodactyly: The role of ultrasonography, superb microvascular imaging, and elastography in diagnosis”	Asymptomatic swelling of PIP and MCP joints with a focus on imaging diagnostics	15/male	1	Skin thickening, lower elasticity, no synovitis or bone changes	Excluded inflammatory arthropathies	Observation	Improved diagnostic accuracy, and reduced need for biopsy	[11]
8	Jubber et al. (2021)	“Pachydermodactyly presenting as juvenile idiopathic arthritis”	Adolescent males misdiagnosed with juvenile idiopathic arthritis	172ah/male	1	Normal inflammatory markers; imaging showed soft tissue swelling only	Juvenile idiopathic arthritis	Diagnosis revised; reassurance provided	Avoided unnecessary medication	[12]
9	Saidy et al. (2020)	“Pachydermodactyly: A case report and review of literature”	Bilateral symmetrical progressive painless swelling of PIP joints with hyperkeratosis	17/male	1	X-ray and MRI showed no inflammatory or malignancy signs; negative serological tests	Rheumatic diseases, connective tissue disorders	Conservative management; observation	Symptoms stable; no functional impairment	[13]
10	Dagrosa et al. (2020)	“Pachydermodactyly associated with extensive computer gaming: A report of three cases”	Three adolescent males with PDD were linked to excessive computer gaming	14–18/males	3	Histopathology showing increased fibroblasts and mucin; imaging with soft tissue thickening	Psoriatic arthritis, knuckle pads, inflammatory arthritis	Behavioral modifications, observation	Symptoms stabilized; no further progression was noted	[14]
11	Liew & ting (2020)	“Pachydermodactyly: A case report of a little-known and benign form of digital fibromatosis”	Bilateral PDD in a male teenager linked to gaming habits	16/male	1	MRI showed subcutaneous thickening; no joint abnormalities	Excluded inflammatory arthropathies	Advised reduced gaming	The swelling subsided over time	[3]
12	Barnes et al. (2018)	“Pachydermodactyly: Case report including clinical and histopathologic diagnostic pitfalls”	Chronic swelling and thickening of PIP joints with lichenified plaques; mimicked arthritis	25/male	1	Soft tissue swelling; normal labs including RF, anti-CCP, ESR; histology showing dermal thickening and mucin	Rheumatoid arthritis, lupus erythematosus, knuckle pads	Behavioral modification; the patient declined intralesional steroids	Stable; no progression of symptoms	[15]
13	Mititelu et al. (2022)	“Two cases of pachydermodactyly presenting as polyarthritis”	Periarticular nodular swelling was initially diagnosed as JIA; and confirmed as PDD post-biopsy	17/male, 14/female	2	Soft tissue swelling without joint involvement; histology showing mucin deposition and coarse collagen	Juvenile idiopathic arthritis, self-healing juvenile cutaneous mucinosis	Hydroxychloroquine, NSAIDs, patient counseling	No significant improvement; symptoms stable	[16]
14	Schneider et al. (2016)	“Pachydermodactyly: A case report including histopathology”	Bilateral swelling over PIP joints; nodular appearance with a history of mechanical trauma	19/male	1	Hyperkeratosis and haphazardly arranged thick collagen fibers; MRI showing soft tissue swelling without bony changes	Juvenile inflammatory arthropathy, connective tissue diseases	Behavioral modification, topical corticosteroids	Significant regression of swelling; stable condition	[17]
15	Equena et al. (2014)	“Case for diagnosis: Pachydermodactyly”	Asymptomatic swelling of PIP joints associated with compulsive finger manipulation	22/male	1	Hyperkeratosis, discrete papillomatosis, mild increase in fibroblasts, and dermal mucinosis	Rheumatoid arthritis, juvenile idiopathic arthritis, synovitis	Behavioral modification, cessation of finger manipulation	Partial regression of lesions; stable outcome	[18]
16	Sagransky et al. (2012)	“Pachydermodactyly from repetitive motion in poultry processing workers”	PDD in poultry processing workers due to repetitive motions	33, 42/males	2	Bilateral PIP swelling; no systemic markers	Occupational repetitive motion injury	Behavioral modification	Reduced symptoms with job modification	[19]
17	Chen et al. (2012)	“Pachydermodactyly: Three new cases in Taiwan”	Three cases of PDD with bilateral PIP joint swelling; two females and one male	13/male, 13/female, 12/female	3	X-rays show soft tissue swelling; no bony changes; normal labs including ANA, RF, and ESR	Juvenile idiopathic arthritis, knuckle pads, Thiemann's disease	Skin care education, avoidance of local trauma	The swelling was reduced with no complications	[20]
18	Ulusoy et al. (2012)	“Unusual unilateral presentation of pachydermodactyly”	A rare familial case of unilateral PDD	16/Male	1	Unilateral PIP joint swelling; no systemic involvement	Familial knuckle pads	Supportive care	Symptoms are nonprogressive	[21]
19	Small et al. (2011)	“A 12-year-old boy presenting with unilateral proximal interphalangeal joint swelling”	Unilateral PDD in a 12-year-old boy	12/male	1	Swelling of PIP joint without systemic abnormalities	Juvenile idiopathic arthritis, inflammatory arthritis	Observation	No progression was noted	[22]
20	Cabanillas et al. (2010)	“Pachydermodactyly in a young girl: a cutaneous manifestation of a psychiatric disorder?”	Adolescent girls with repetitive behavioral habits linked to PDD	15/female	1	Soft tissue thickening on PIP joints without systemic findings	Behavioral-linked PDD	Behavioral therapy	Symptoms reduced after habit cessation	[23]
21	Taylor-Gjevre et al. (2009)	“A case of deforming pachydermodactyly”	Atypical deforming PDD with subluxation of the interphalangeal joint in an adult male	39/male	1	Nonerosive subluxation seen on imaging, normal inflammatory markers	Inflammatory arthropathy (e.g., psoriatic arthritis)	Supportive management	Persistent but nonprogressive symptoms	[5]
22	Park et al. (2006)	“A case of pachydermodactyly treated by surgical excision”	Surgical excision for a patient with significant PDD-related swelling	22/male	1	Subcutaneous thickening was confirmed histologically	None specified	Surgical excision	Symptoms resolved	[24]
23	Woodrow et al. (2003)	“Pachydermodactyly in association with Asperger syndrome”	Young adult with Asperger syndrome presenting with bilateral PIP joint swelling	15/male	1	No systemic or inflammatory markers; MRI revealed subcutaneous thickening	Inflammatory arthritis	Addressed underlying psychiatric condition	Swelling is reduced with reduced repetitive behavior	[25]
24	Yüksel et al. (2023)	Pachydermodactyly	Symptomatic PDD in a young male with anxiety issues and repetitive finger friction behavior	15/male	1	Symmetrical soft tissue swelling over the lateral aspects of 2^nd^ to 4^th^ PIP joint bilaterally Normal inflammatory markers MRI: sof tissue swelling	—	Behavioral modification and psychological support	—	[26]

## Data Availability

The data that support the findings of this study are available in the Supporting Information of this article.
